# Eye movements when viewing advertisements

**DOI:** 10.3389/fpsyg.2014.00210

**Published:** 2014-03-17

**Authors:** Emily Higgins, Mallorie Leinenger, Keith Rayner

**Affiliations:** Department of Psychology, University of CaliforniaSan Diego, CA, USA

**Keywords:** advertising, eye movements, visual attention, saccades, marketing

## Abstract

In this selective review, we examine key findings on eye movements when viewing advertisements. We begin with a brief, general introduction to the properties and neural underpinnings of saccadic eye movements. Next, we provide an overview of eye movement behavior during reading, scene perception, and visual search, since each of these activities is, at various times, involved in viewing ads. We then review the literature on eye movements when viewing print ads and warning labels (of the kind that appear on alcohol and tobacco ads), before turning to a consideration of advertisements in dynamic media (television and the Internet). Finally, we propose topics and methodological approaches that may prove to be useful in future research.

Eye movements are of interest, with respect to viewing advertisements and more generally, because they provide fine-grained information about patterns of visual attention. Because we cannot process detailed information far beyond the *fovea*, the central region of the retina spanning about 2° of visual angle, we must move our eyes from one location to the next, sequentially *fixating* (or looking directly at) areas of interest (Rayner, 1998, 2009). Saccade targets are determined, in large part, by our immediate cognitive or perceptual requirements. Eye movements are thus an important way in which we exercise active selection over our complex visual environments (Findlay and Gilchrist, 2003). By inspecting the eye movement record we can, consequently, make inferences about how viewers selectively attend to the visual world, whether they are reading, viewing natural scenes, searching for a target item, or, as is of primary concern here, viewing advertisements.

It is important to note, at this point, that *eye position* and the *locus of visual attention* are not precisely identical concepts, since it is possible to disengage attention from the current point of fixation (Posner, 1980). Indeed, our attention generally shifts to the *next* location we will fixate shortly before we actually move our eyes (Rayner et al., 1978; Kowler et al., 1995; Deubel and Schneider, 1996). However, attention and eye movements are typically quite closely coupled (and, when they do become separated, it is generally in the systematic manner just described, so that the eyes will soon “catch up” with the focus of attention). Therefore, fixation distributions provide detailed information about which regions of a display most effectively capture visual attention. Furthermore, the duration spent fixating each location provides information about the amount of cognitive and perceptual processing devoted to that region (Rayner, 1998, 2009).

Research on eye movements and advertisements can provide general theoretical insights (Rayner et al., 2001; Wedel and Pieters, 2008b). For instance, the domain is well-suited for investigating the relationships between eye movements and higher-level phenomena, such as memory and preference. Furthermore, work in this area can shed light on how we integrate text and images as we inspect our visual environments, as ads are often complex stimuli, composed of both elements. As Buswell (1935) noted in his classic study of eye movements and scene perception, this research may be also be useful from an applied perspective (see Duchowski, 2002 for a general review of applied eye movement research).

There are several reasons why eye tracking may be useful to those who design advertisements or public policy notices such as warnings on alcohol and tobacco products. First, eye movements can provide insight into the fast and detailed dynamics of visual attention that may simply not be available for introspection or verbal report (Pieters and Wedel, 2008). Second, eye tracking can be done in real time during ad viewing without interfering with ongoing processing (Russo, 1978; Wedel and Pieters, 2008a; Glaholt and Reingold, 2011). Third, the technique seems less prone to biasing subsequent responses of interest (e.g., choice of product or brand memory) than verbal protocols. Fourth, eye tracking can provide an efficient means of pinpointing which specific characteristics of an ad contribute to its success or failure in holding viewers’ attention or driving consumer choices^1^. Of course, the technique is limited with regard to the kinds of information it can provide: if a researcher or advertiser were primarily interested in viewers’ conscious, emotional reactions to a given image, for example, soliciting verbal responses would be preferred. Used in conjunction with other approaches, however, including interviewing subjects, testing their memory for products or brands, and tracking their selections, the technique can contribute substantially to applied research on advertisements (Treistman and Gregg, 1979).

We begin by providing some background information on the basic properties of eye movements as well as their characteristics in reading, scene perception, and visual search. These topics are relevant because ads often consist of both text and scene-like information, and may also include a search component (if, for example, one is searching in a supermarket circular for a particular product of interest). Next, we will provide a more specific review of key findings concerning eye movements when viewing advertisements, including print ads, warning labels, and ads appearing on television (TV) and on the Internet^2^. Finally, we outline some topics that have, up to this point, remained relatively unexplored, as well as methodological approaches that may prove useful in future research.

## BACKGROUND INFORMATION ON EYE MOVEMENTS

### BASIC CHARACTERISTICS

While we can produce several different types of eye movements (see Rayner, 1998 for a review), only *saccades* are covered here, since they are most critical for the research reviewed. Saccades are fast, darting movements that we perform about three times each second (Schiller, 1998). They are interleaved with brief periods of relative stability, known as *fixations*, which last on average about 200–300 ms, depending on the task and the individual (Rayner, 1998, 2009). Saccades can reach velocities as high as 500° of visual angle per second. While their duration is dependent on the distance covered and varies as a function of task, they generally last about 20–50 ms. During these movements, effective visual processing is largely suppressed (Matin, 1974; Campbell and Wurtz, 1978), such that useful visual information can only be gathered during the intervening fixations.

Saccades are executed, as was noted above, in order to bring the fovea, the central 2° of the visual field with high acuity and good color vision, into alignment with the region we wish to process. The region surrounding the fovea and extending up to 5° of visual angle from fixation is known as the *parafovea*, while the region that lies beyond the parafovea is known as the *periphery* (note, however, that acuity drops off in a continuous fashion with increasing distance from the fovea, so that no sharp distinction should be drawn between the parafovea and periphery; Liversedge and Findlay, 2000). Although we make use of the lower resolution, parafoveal and peripheral information (e.g., to begin to process an upcoming word when reading or to decide where to move the eye next), for most tasks requiring the rapid processing of detail, foveal processing is necessary (Rayner, 1998, 2009).

### NEURAL BASIS OF SACCADE TARGETING

The neural underpinnings of saccade targeting span multiple cortical and sub-cortical structures involved in attention, visual processing, and motor planning. We present a brief overview of some of the important aspects of this system here (for reviews, see Gaymard et al., 1998; Schiller, 1998; Liversedge and Findlay, 2000; Pierrot-Deseilligny et al., 2004; Schall and Cohen, 2011).

A saccade occurs when the extraocular muscles, arranged in three opposing pairs around the eye, are appropriately stimulated by premotor structures in the brainstem. Regions of the superior colliculus (SC), located in the midbrain, are critical for controlling these saccades. One population of cells in the SC fires continually during fixation, ceasing to fire just before a saccade is executed and remaining inactive for much of the duration of the saccade. Another population of cells forms a map of the visual field. The level of neural activity at different locations in the map appears to code for the importance of the corresponding locations in the visual scene. Thus, this population of cells is sometimes referred to as a *salience map*, with areas of high activity (or “peaks”) marking important positions that serve as candidate targets for the upcoming saccade (Findlay and Gilchrist, 2005).

Similar maps appear to exist in other, cortical areas of the brain that project to the SC, though they are sometimes known as *priority maps* in these higher areas (Schütz et al., 2011). Maps in a region of the frontal cortex known as the frontal eye fields (FEF) may be important for directing endogenous, or top-down, saccades – i.e., saccades based largely on the goals of the viewer^3^. In contrast, the parietal eye fields (PEF) in the parietal lobe appear to be particularly important for coding exogenous, reflexive, or bottom-up saccades, of the kind that might occur, for example, following the sudden onset of a stimulus. Other frontal regions may be involved in suppressing such saccades, however, when executing them would be undesirable for present purposes (Pierrot-Deseilligny et al., 2004).

Notably, when mild stimulation, insufficient to trigger a saccade, is applied to the SC or FEF, this leads to superior visual processing at the corresponding locations in the scene (see Noudoost et al., 2010 for a summary), indicating overlap between the visual attention system and the oculomotor system (see Desimone and Duncan, 1995 for a review of visual attention in the brain).

While the basic principles of the oculomotor system hold true across tasks, it is important to note that eye movement measures in one task (e.g., reading) can differ substantially from those in other tasks (e.g., scene perception). This likely follows from differences both in the physical stimuli involved and in the nature of the viewers’ goals and cognitive processing across these different activities. Therefore, we outline the basic characteristics of eye movements during reading, scene perception, and visual search below.

### READING

When reading, fixations tend to be on the order of 225–250 ms. Average saccade length is seven to nine letters in alphabetic languages (Rayner, 1998, 2009). For speakers of English, and other languages written from left to right, most eye movements proceed in that direction, with *regressions* (i.e., saccades that move backward in the text) representing 10–15% of eye movements. Readers only fixate about 70% of the words in the text, *skipping* the other 30%.

Eye movements during reading provide an online index of the cognitive processes underlying language comprehension: in fact, how long the eyes remain fixated on a given word largely depends on how easy or difficult it is to process. Lexical variables such as word frequency and predictability have strong influences on fixation durations (for reviews, see Rayner, 1998, 2009), as does reading skill (Ashby et al., 2005) as well as typographical factors such as font difficulty (Rayner et al., 2006; Slattery and Rayner, 2010).

Though a large amount of text falls on the visual field during reading, readers are only able to obtain useful letter information from approximately 18–20 character spaces around fixation, and they do not use information from lines above or below the currently fixated line (Inhoff and Briihl, 1991; Inhoff and Topolski, 1992; Pollatsek et al., 1993). This limited area of effective processing, known as the *perceptual span*, is asymmetrical in the direction of upcoming text (and attention), such that, for readers of English, it extends about three to four character spaces to the left of fixation (McConkie and Rayner, 1976; Rayner et al., 1980) and 14–15 characters to the right of fixation (McConkie and Rayner, 1975; Rayner and Bertera, 1979).

While fixation location and visual attention coincide when we are processing a fixated word, they may become decoupled when processing of that word is complete. While the eyes remain fixated on the current word, attention can nonetheless shift to the upcoming word (located parafoveally, but within the perceptual span) so that processing of this parafoveal word can begin. This preprocessing prior to actual fixation will facilitate foveal processing following a saccade to that word, giving rise to a *preview benefit*. Preview benefit is measured using a gaze-contingent boundary paradigm (Rayner, 1975), in which an initial preview of a target word is replaced with the word itself when the subject’s eyes cross an invisible boundary during the saccade to the target (note that, because the display change occurs during the saccade, when vision is largely suppressed, subjects generally fail to notice it; Slattery et al., 2011). The preview may be identical to the target or may be a non-identical letter string. During reading, this preview benefit, defined as the reduction in foveal viewing time of the target following an identical vs. a non-identical preview, is about 30–50 ms (for reviews, see Rayner, 1998, 2009; Schotter et al., 2012).

### SCENE PERCEPTION

During scene perception, viewers make both longer fixations and longer saccades than when reading text. Fixations last, on average, about 300 ms, while saccades span approximately 4–5° of visual angle (though both figures vary depending on the specific features of the scene as well as the task at hand). Furthermore, the perceptual span in scene viewing is substantially larger than in reading, though its precise extent is not as well understood as it is in reading (Rayner and Castelhano, 2008; Rayner, 2009). In addition, just as in reading, viewers obtain a preview benefit during scene perception (Pollatsek et al., 1984, 1990; Henderson et al., 1987 1989; Henderson, 1992; Henderson and Siefert, 1999, 2001). The magnitude of this benefit appears to be on the order of 100 ms (Rayner, 1998, 2009).

Within our very first fixation on a scene we are, rather impressively, able to extract its global meaning or *gist*, distinguishing, for example, an indoor from an outdoor scene or a forest from a mountain landscape (Henderson, 2003; see Oliva, 2005 for a review of gist processing). This first glimpse is thought to orient the viewer and provide some guidance about subsequent eye movements (Rayner, 2009). When viewers do go on to inspect the rest of the scene, they do not fixate all regions with equal probability. Rather, they tend to selectively view those elements that are particularly meaningful or relevant. For instance, viewers inspecting a scene of two figures walking in a garden would devote a great many more fixations to the people’s faces than to a nearby patch of plain grass (see Buswell, 1935 for a classic demonstration of this effect). In addition, if a region is visually distinctive or *salient* – for example, if it is of higher or lower intensity than its immediate surroundings – it will tend to draw a disproportionate number of fixations (Parkhurst and Niebur, 2003).

The goals of the viewer also affect eye movements during scene perception. Yarbus (1967), for instance, found that viewers inspected a single painting, Repin’s *The Unexpected Visitor*, quite differently depending on their instructions. In the painting, a man (the “visitor”) enters a domestic scene. When viewers were asked to decide how long the visitor had been away, for instance, fixations seemed to cluster mainly on the faces of the individuals in the room. When asked to determine the economic circumstances of the family depicted, however, viewers’ fixations appeared more widely dispersed, landing more upon objects in the room (such as pieces of furniture or clothing) that might provide information about prosperity than in the former condition.

Finally, one striking finding regarding scene perception is that, despite the common intuition that we monitor our visual environments quite closely (Levin et al., 2000), research indicates that we may miss even rather dramatic changes provided that they happen during a saccade or other visual disruption. Grimes (1996; see also McConkie and Currie, 1996), for example, investigated subjects’ sensitivity to dramatic changes in natural scenes introduced during saccadic eye movements. Even with prior warning that such changes might occur, subjects’ ability to detect them was surprisingly limited. For example, when a flock of birds in one scene dwindled in number by about a third during an eye movement, subjects reported noticing something odd only about 10% of the time. Importantly, however, if the changing object is pre-cued (Rensink et al., 1997) or lies near the target of the critical saccade (i.e., the saccade during which the change occurs), change detection rates improve (Henderson and Hollingworth, 1999). These findings highlight the critical role of attention in determining how we perceive our visual environments.

### VISUAL SEARCH

Visual search is an important part of many everyday activities. We perform such searches, for example, when looking for tea at the grocery store or trying to find our keys on the way to work each morning. The basic parameters of fixations and saccades during visual search are quite variable. Overall, average fixation times are reported to be between 180 and 275 ms, while average saccade size tends to be intermediate between that of reading and that of scene perception, but can vary widely (Rayner and Castelhano, 2008). Such variability is perhaps to be expected since, as will be seen below, eye movement patterns during search exhibit a remarkable flexibility and sensitivity to the specific demands of the moment.

When we search for an item of interest, both bottom-up (or stimulus-driven) and top-down (goal-driven) factors guide our eye movements. Bottom-up guidance is evident when eye movements are drawn to a region that stands apart from its surroundings, irrespective of the qualities of the search target (see Itti and Koch, 2001 for a review of models that emphasize bottom-up effects on attention and eye movements). An item that stands out in a highly salient manner from all surrounding objects (e.g., a single tilted line amid a field of vertical lines) is said to “pop out” (Wolfe, 1994).

Top-down guidance is driven by the properties of the target and their relationship with various elements of the scene. For instance, if we are searching for a bright yellow car in a crowded parking lot, similarly bright cars will preferentially attract our eye movements (Pomplun, 2006). When we perform *conjunctive* visual search, i.e., search for a target that is defined by a pair of properties (e.g., being both round and red), fixations cluster preferentially on items belonging to the less frequent property in the display (Shen et al., 2003). This illustrates the remarkable sensitivity of our eye movement system to the relative informativeness of different stimulus features during search.

Top-down search also operates when our high-level expectations about where a target object is expected to reside affect search behavior. For instance, when searching for a computer monitor in an office scene, eye movements will cluster on the desk, rather than along the floorboards (Neider and Zelinsky, 2006). In general, recent research suggests that, while bottom-up guidance plays a role in search, top-down guidance may be dominant during real-world search for meaningful objects (e.g., Chen and Zelinsky, 2006; Pomplun, 2006; Henderson et al., 2007; Peters and Itti, 2007).

## VIEWING ADVERTISEMENTS

We now turn to examine research more specifically focused on eye movements when viewing advertisements. We discuss print advertisements, warning labels, and dynamic media (TV and the Internet) in turn.

### PRINT ADVERTISING

Viewers obtain the gist of print advertisements very quickly, reliably discriminating them from editorial content – and, under some conditions even identifying the advertised product – after exposures of only 100 ms (Pieters and Wedel, 2012). In this section, we examine some of the factors that guide attention after this initial glimpse, as viewers begin to actively explore advertisements by shifting their gaze from one location to the next within the display. We begin by considering the composition of ads, including basic visual properties (e.g., color and size) as well as higher-level, semantic cues. Next, we review effects of ad originality (or creativity) as well as repetition. We then consider how viewers’ goals or tasks affect viewing behavior before turning, finally, to briefly review findings concerning the integration of text and picture processing when viewing print advertisements. At several points throughout the review, the relationship between eye movements and higher-level phenomena such as memory will also be discussed.

#### Ad composition

In this section, we review critical findings on the relationship between the composition of print ads and eye movement measures. We begin by examining possible effects of basic, visual characteristics and then proceed to a consideration of higher-level, semantic aspects of advertisements.

Lohse (1997) tracked subjects’ eye movements as they viewed yellow page advertisements and selected products from various categories as if for purchase. Viewers were more likely to look at large ads than small ads (see also Pieters et al., 2007), though small display ads received more fixations per unit area than large display ads (see Peschel and Orquin, 2013 for a review of surface size effects on visual attention). Viewers were also more likely to fixate on color than black and white ads, and looked at color ads sooner (i.e., nearer the beginning of the fixation sequence) and for a longer duration. In addition, they spent marginally more time viewing ads that contained pictures than those that did not. The location of the ad was also important, such that ads near the end of the page were often skipped. Products that were subsequently selected also received considerably more visual attention than did those that were not. Lohse and Wu (2001) conducted a similar study, this time presenting a directory in Mandarin to Chinese subjects and replicated the main findings of the original study, suggesting that these effects are not culturally specific.

Other research has examined possible effects of the size of particular elements of advertisements, such as the text or picture, on patterns of visual attention. When ads were presented as part of a competitive visual array (as in a supermarket circular), Pieters et al. (2007) found that ads with larger pictures, but not larger text elements, were more likely to be fixated and were viewed for longer. In contrast, Pieters and Wedel (2004) found that when subjects inspected solitary advertisements in magazines, ads with larger text elements, but not larger pictures, were more likely to be fixated and viewed for longer. (The *presence* of a picture, however, independent of its size, did appear to attract attention under these conditions.) Comparing these findings may suggest that sufficient picture size is particularly important for capturing and holding attention in competitive visual environments, while a sufficient amount of text may be especially important when ads are presented alone. However, the results were obtained in separate studies using stimuli that differed in several respects (e.g., types of product advertised, the range of text and picture sizes), so no strong claim to that effect can yet be made.

Interesting findings have also been reported regarding brand elements (e.g., logos) of advertisements in particular. While intuition might suggest that viewers will be repelled by them, since they serve as a salient reminder that the stimulus is an ad rather than a piece of editorial content, some eye movement data suggest otherwise. First, Wedel and Pieters (2000) found that, among all ad elements, the brand received most fixations per unit of surface area (but see Ryu et al., 2009). Second, each fixation on the brand element predicted a greater improvement in performance on a subsequent recall test than did each fixation on the text or pictorial^4^. Third, increasing the size of the brand element did not reduce overall viewing times on ads, as one might expect on the theory that salient brand elements reduce attention to advertisements (Pieters and Wedel, 2004). However, as will be noted below, the sustained presence of a central brand element in TV commercials is associated with ad skipping (Teixeira et al., 2010).

Visual competition or clutter, an issue of considerable importance in many visually complex contemporary environments, has also been examined. Pieters et al. (2010) found that high levels of visual feature complexity in advertisements was associated with reduced viewing of the brand element. Visual competition is also a concern when designing “feature advertisements” (such as supermarket circulars), wherein multiple ads are displayed simultaneously and must compete for viewers’ attention. Janiszewski (1998) found that items subject to greater visual competition by surrounding objects were viewed for less time and, in a separate experiment, remembered less well than items subject to less competition.

Janiszewski (1998) also proposed that the layout of feature advertisements could be optimized (from the perspective of the advertiser), without removing any items, in order to minimize visual clutter and maximize overall viewing time. Pieters et al. (2007) extended this line of inquiry, developing a model to minimize visual competition (based on the Attention Engagement Theory; see Duncan and Humphreys, 1989, 1992). This optimized layout led to an increase in overall viewing time of the entire ad array when compared with the existing layout. Average time spent viewing a particular feature ad, given that it was fixated, was also higher in the optimized layout, though average probability of fixating an ad within the array declined. Furthermore, Zhang et al. (2009) developed a Bayesian model that, they argue, suggests that the layout of feature advertisements can affect sales and that this effect is mediated by visual attention on ads. However, confounds are, of course, a concern in correlational research of this kind (though Zhang et al., 2009 adopted a statistical approach designed to circumvent several concerns of this nature).

Simola et al. (2013) examined both the semantic and the spatial relationships between ads and editorial material. They found that when the semantic content of ads was congruent with the text – for instance, a beer ad accompanying an article about beer – these ads were (at least when presented on the right) remembered better than were incongruent ads. Interestingly, however, incongruent ads received more visual attention (also when presented on the right) than did congruent ads (but see Hervet et al., 2011, discussed below). This difference only appeared in “second-pass” viewing of the ad (that is, on a return to the ad after having left it), suggesting that an initial fixation on the ad was required before effects of semantic congruency could influence eye movements. Simola et al. also found that ads received more visual attention and were recognized better when placed to the right of the editorial content.

Social cues contained within advertisements have also been examined. Hutton and Nolte (2011) recently demonstrated, for instance, that when a model in an advertisement looks at the product on display, rather than looking forward toward the viewer, subjects spend longer inspecting the product, the brand logo, and the advertisement as a whole.

Classic research has also found that the presence of a human form may affect viewing behavior (Nixon, 1925; see also (Kroeber-Riel, 1979) citing a study by Witt, 1977 concerning the level of undress exhibited by a figure in an advertisement). Research in scene perception indicates, however, that when attempting to discover effects of high-level, semantic aspects of a stimulus, it is important to control for possible differences in low-level visual salience (see Rayner, 1998 for a discussion of such considerations). Future research could build upon these early studies, then, by determining and attempting to control for differences in low-level visual salience across ads, thus allowing us to draw stronger inferences about the possible role of these higher-level, semantic factors.

#### Originality

When ads are particularly creative or original, how do viewers respond? Radach et al. (2003) compared viewing behavior, affective responses, and memory for “implicit” and “explicit” ads. The explicit ads featured text and images that were related to one another and to the product being advertised in a fairly straightforward manner while, in the implicit ads, these relationships were more creative and less direct. The implicit ads were viewed for longer than their explicit counterparts and, while mean fixation duration and saccade amplitudes did not differ across ad types, the implicit ads received significantly more fixations than did the explicit ads. Subjects also liked the implicit ads better than the explicit ones and rated them to be more interesting than their explicit counterparts^5^. Overall, memory for the implicit and explicit ads was similar, but a detailed analysis suggested that there might have been a slight advantage for the implicit ads in some conditions (see also Pieters et al., 1999b).

However, Pieters et al. (2002) pointed out that while consumers like original ads and view them for longer periods overall, they may attend selectively to the particularly creative or artistic aspects of the advertisements, potentially at the expense of the brand or product advertised. Thus, while such creative ads may please the viewer, they may not serve the interests of the advertiser if, indeed, they direct attention away from the advertised brand. Pieters et al. conducted an experiment that partially addressed this question by comparing viewers’ fixations on the brand elements (such as the logo) of original or creative ads with more typical ads. Brand elements in the creative ads tended to receive *more*, not fewer, fixations than those of their typical counterparts, suggesting that creative ads may not, in fact, divert attention from the advertised brand, but rather may serve to increase it.

#### Repetition

Another potentially important factor in real-world ad viewing is that a viewer may well be exposed to a particular ad repeatedly (if, for instance, it runs in multiple magazines). Pieters et al. (1996) addressed this topic, finding that when subjects were exposed to an ad three times over the course of an experimental session, viewing time decreased with additional exposures (see also Pieters et al., 1999a). More elements of the ad were also skipped in the third than in the first viewing. Furthermore, an effect of subject motivation on viewing time (to be described below) disappeared by the third exposure. Pieters et al. (1999a) maintained, however, that the probabilities of moving from each ad element (e.g., the headline) to each other element (e.g., the pictorial) on the next fixation remained stable over repeated exposures (see also Rosbergen et al., 1997b). It is not yet clear, however, how well each of these findings will generalize to (arguably more naturalistic) conditions in which exposures to the ad are spaced out over longer intervals.

Finally, Pieters et al. (2002; see also Pieters et al., 1999b) investigated the eye movement patterns associated with ads of varying prior familiarity. Ads rated as being more familiar (by trained raters not participating in the eye movement study) were fixated less frequently than were less familiar ads. The effect seemed mainly to be driven by a decline in fixation frequency on the text with increasing ad familiarity. However, if an ad was particularly original or creative, this ameliorated negative effects of familiarity.

#### Goals

As was discussed above, top-down factors concerning the viewer’s goal have long been known to affect eye movement behavior during scene perception and other visual activities. More recent research has also examined effects of goal or task when subjects view advertisements and has demonstrated that these factors can have a profound effect on viewing behavior.

Perhaps unsurprisingly, when subjects control viewing time, they inspect ads for longer when given instructions that encourage deeper processing. An important implication of this general finding (to be discussed in more detail below) is that viewing behavior during laboratory tasks that promote deep engagement with advertisements is likely to differ substantially from real-world ad viewing, which is often quite cursory (Wedel and Pieters, 2000; Pieters and Wedel, 2004, 2007, 2008).

Pieters et al. (1996) compared behavior in a “high motivation” condition, in which subjects were instructed to view ads carefully and told they would later be allowed to select one of the advertised products, to that in a “low motivation” condition, in which subjects were simply told to evaluate the “draft versions” of the ads (see also Pieters et al., 1999a, Study 2). In early exposures to the ad, highly motivated subjects viewed ads for substantially longer, although, as was noted above, this difference disappeared by the third exposure. Similarly, Rayner et al. (2001) compared viewers’ responses to “critical” ads, those featuring a product to be evaluated as if for purchase, and “non-critical” ads, featuring products from another category. Critical ads were fixated more and viewed for significantly longer than were non-critical ads. Critical ads were also missed less, in a subsequent recognition memory test, than were non-critical ads (though no such advantage for critical items appeared in a free recall test). In addition, Radach et al. (2003) found that when subjects were asked to decide how much they liked an ad, they viewed it for substantially longer than when they were asked to paraphrase the message of the ad. Subtle differences in task, however, may not be sufficient to drive this effect, as Rayner et al. (2008) found no significant differences in total ad viewing time when subjects were instructed to evaluate an ad for its effectiveness or decide how much they liked it.

The total time spent viewing an ad (presented in isolation) can, of course, be measured perfectly well without eye tracking. However, eye movement data can also reveal more fine-grained differences across tasks. In particular, some eye tracking research suggests that viewers’ goals affect the proportion of time they allocate to different ad elements, such that tasks that require considering the brand or product advertised in a fairly deep manner may favor the text, while tasks that encourage more shallow processing, or making judgments about the quality of the ad itself, may favor picture viewing.

First, Radach et al. (2003) found that when subjects were asked to evaluate an advertisement, they viewed the picture longer than the other components and subsequently recalled more information about the picture. When subjects were asked to paraphrase the message of an ad, however, viewing time on the picture substantially declined. In addition, Pieters et al. (1996) found interesting differences in text and picture viewing between high and low motivation conditions. However, the effects were only significant in the second of three presentations of the ad, so they should perhaps be viewed as tentative at this time. In the second exposure to an advertisement, low motivation subjects spent a greater proportion of time viewing pictures than did those in the high motivation group. Conversely, high motivation subjects spent a greater proportion of time viewing the text than low motivation subjects.

Pieters and Wedel (2007; see also Wedel et al., 2008 for further analyses of these data) also found that body text and picture viewing were affected differently by task. Subjects spent most time viewing the text in a task that required subjects to learn about the advertised brand. In contrast, viewers’ eye movements were drawn preferentially to the picture in conditions that required subjects to memorize the ad or view it freely as they would at home.

Comparing the findings of Rayner et al. (2001), in which subjects were instructed to consider one of the types of advertised products for purchase, and Rayner et al. (2008), wherein subjects made judgments about the ads themselves (whether they liked them and how effective they were) also suggests that different goals may affect text and picture viewing patterns differently. In Rayner et al. (2001), text elements were viewed for a great deal longer than the pictures, while in the latter study, the pictures were viewed longer than the text (though the effect failed to reach statistical significance in an analysis that controlled for differences in surface area across elements). Furthermore, early looks tended to be drawn toward text in the 2001 study (on average, the text was reached by the third fixation) but toward the picture in the 2008 study.

Rayner et al. (2008) compared data obtained in the two experiments, considering only the subset of stimuli that were used in both. Based upon this analysis, they suggested that differences in subject instructions did likely contribute, to some extent, to the differences in viewing behavior across studies. This interpretation should not be viewed as conclusive, however, since the data compared were collected in separate experiments. It should also be noted that, when text and picture viewing for critical and non-critical ads were compared within the Rayner et al. (2001) study, no clear interaction of the expected type (i.e., showing a text advantage for critical ads and a picture advantage for non-critical ads) emerged^6^.

Rosbergen et al. (1997a) obtained related results using latent class analysis to segment viewers into three distinct groups. While task was not manipulated in this study, subjects’ attitudes about the advertised products were recorded and compared with the eye movement data. The picture (as well as the headline) was favored by the subject group who spent the least time viewing the ad overall and deemed the advertised product to be particularly low in risk (i.e., they thought that choosing incorrectly would not be a costly error; Jain and Srinivasan, 1990, as cited in Bearden and Netemeyer, 1999). The only group to spend a substantial portion of the time viewing the body text was that which spent the most time viewing the ad overall, perhaps indexing deeper consideration of the advertised product. Additionally, subjects in this group viewed the product as more risky than did those in the other groups. Overall, then, the evidence suggests that deep engagement with the product advertised (and its attendant risks) may bias subjects toward the text, while more casual viewing, or evaluation of the advertisement itself, may bias viewers toward the picture.

#### Integrating text and picture viewing

We now consider research on how viewers integrate text and picture elements while inspecting print ads. Rayner et al. (2001) found that average fixation duration when viewing the picture in an ad (about 266 ms) was significantly longer than when viewing the text (about 226 ms). Viewers also made longer saccades on average (about 4.5° of visual angle) when examining a picture than when reading the text (about 3.1°). These findings were replicated in Rayner et al. (2008) and are also quite consistent with the broader literature on differences in eye movements when viewing text and pictures (Rayner and Castelhano, 2008).

Rayner et al. (2001, 2008) also found that viewers generally did not quickly alternate between fixating the text and the picture but rather tended to remain on one component or the other for several fixations in a row. More specifically, given that a fixation was on the picture, the next fixation would also be on the picture about 78% of the time; if a fixation was on the text, the following fixation would remain on the text about 77% of the time (Rayner et al., 2008). Pieters et al. (1999a) reported similar findings.

However, Radach et al. (2003) reported (somewhat informally) that viewers tended to look back and forth fairly frequently between different elements of the ad, including the text and the picture. They suggested that this may have been due to the relatively high demands placed on subjects in their study. Indeed, as we have seen, the goal of the viewer can substantially affect viewing behavior. However, another possibility is that the nature of the stimuli, and in particular the text used within the ads, may have differed across experiments. In particular, many of the ads used by Rayner et al. contained somewhat lengthy passages of “body text.” If the stimuli used by Radach et al. (2003) contained shorter snippets of text (in the form of headlines or brief slogans), one might imagine that this could lead to more alternating between text and pictures if readers adopted a “sampling” approach rather than a reading approach toward the text. This idea is, of course, purely speculative, but it could be tested experimentally in future research.

In summary, then, a number of factors appear to guide eye movements when viewing print advertisements. These include size, color (Lohse, 1997; Lohse and Wu, 2001), and visual clutter (Janiszewski, 1998), as well as higher-level social cues, such as the direction of a model’s gaze (Hutton and Nolte, 2011). Creative or original ads are also fixated more than typical ads, and are liked better, and deemed more interesting (e.g., Radach et al., 2003). Repeated exposures to a given ad reduce viewing times, at least when these exposures occur in short succession (Pieters et al., 1996). However, the transition matrices between ad elements, indexing the probability of making a saccade from one element to another, remain fairly stable across multiple viewings (Pieters et al., 1999a). In addition, the beneficial effects of a particularly creative ad may ameliorate the negative influences of repetition (Pieters et al., 2002). The goal or task of the viewer also strongly influences how long we view ads (e.g., Rayner et al., 2001) and may, furthermore, change the proportion of time spent viewing specific ad elements (such as the text vs. the picture). Research on eye movements when viewing text and pictures in ads mirrors the broader eye movement literature in that both fixations and saccades are longer when viewing pictures than when reading text (Rayner et al., 2001). Somewhat mixed findings have emerged on the question whether viewers tend to skip back and forth between text and pictures or remain on one element for a more extended period (compare Radach et al., 2003 with Rayner et al., 2001, 2008). However, two possible explanations for these discrepancies have been proposed (one concerning differences in task and the other concerning differences in stimuli), and future research may resolve this question. Finally, in some of the studies reviewed, eye movement measures were correlated with subsequent measures of memory for the advertised product or brand. In the upcoming sections of the article, reviewing eye movements when viewing warning labels as well as ads presented on TV or the Internet, we will continue to explore issues of eye guidance, as well as the relationship between eye movements and higher-level phenomena such as memory.

### WARNING LABELS

When studying how viewers inspect advertisements, we are often interested in what elements of an ad capture and hold viewers’ attention. While most information (pictorial or textual) is redundant in its attempt to persuade consumers and provide them with a favorable impression of the advertised product or brand, there is one clear-cut exception. The inclusion of health warnings on alcohol and tobacco advertisements represents a clear case in which the information gleaned from viewing the advertisement varies as a function of which regions are viewed.

Across several studies investigating the viewing of alcohol and tobacco warning labels, the general finding is that these labels are often never viewed, and when they are viewed, it is for a very small percentage of the overall ad viewing time (e.g., Fischer et al., 1989; Fox et al., 1998; Thomsen and Fulton, 2007). Because, in the United States, these warnings are usually small in relation to the overall advertisement (taking up, for example, only 3.2% of the ad in a sample used by Fischer et al., 1989), entirely text-based, and black and white, they are unlikely to capture and hold viewers’ attention. Multiple lines of research have therefore investigated the viewing time and recall of warning labels in existing advertisements and compared them with those in which the salience of the warnings has been manipulated.

In one of the first such studies, Fischer et al. (1989) recorded the eye movements of adolescents viewing real cigarette and alcohol advertisements. They found that on 43.6% of trials, subjects never directly fixated the warning, and that on 19.8% of trials subjects looked at, but did not read the warning^7^. On average, subjects looked at the warning labels for only 750 ms, which corresponded to 8% of the total ad viewing time, and this time was unaffected by differences in content, position, or shape (though the stimulus set was small – only five advertisements were tested). Additionally, they found that performance in a subsequent masked recall test of warning label content (where subjects were shown the original ad with the warning label and other areas masked and asked to recall the content) was positively correlated with both mean looking and reading time.

To investigate the effects of various cues on attentional capture and ease of identification, Laughery and Young (1991) manipulated the saliency of warning labels by including pictorials, icons, colors, borders, or combinations of these four cues, and measured the time it took subjects to locate the warning label (i.e., the time from image onset to the first fixation on the warning label), as well as the time it took them to determine that the information was a warning (measured by the time from first fixation on the label until a button was pressed). Time to locate the warning was numerically shorter when any of the saliency manipulations were included, and significantly shorter when the pictorial cue, the color cue, or all four cues combined were included. Similarly, the time to determine that the label was a warning was significantly shorter when a pictorial was included, either alone or combination with other cues. However, since the subject’s goal was to determine whether or not a warning was present in each advertisement, the procedure was, in fact, a visual search task. Thus, it is unclear whether the results would generalize to a more naturalistic, passive viewing of advertisements.

To answer this question, Krugman et al. (1994) compared the eye movements of subjects viewing ads with standard, federally mandated cigarette warnings to novel warnings, which were the same size and shape, but could differ in text, color, graphics, and print type. To keep ecological validity high, the subjects were asked to view the advertisements as they would in a magazine. Novel warnings attracted more attention (i.e., were fixated by more subjects) and attracted attention sooner (i.e., were fixated more rapidly) than the standard warnings. Additionally, Krugman et al. (1994) found that the time spent viewing the warning was positively correlated with masked recall performance for content of the new ads (note that they did not measure masked recall of the standard ads because of subject familiarity).

More recently, Thomsen and Fulton (2007) examined the eye movements of adolescents viewing alcohol ads with moderation messages (e.g., “drink responsibly”). They found that, on average, subjects only fixated the moderation message for 350 ms, which corresponded to 7% of the total viewing time, and that in 75% of the ads with small moderation messages, that message was the least fixated area of the advertisement. However, when the moderation message was a central theme, subjects viewed the message significantly longer (on average 710 ms, compared to 170 ms when the message was not a central theme). In general, recall for even general concepts of the moderation messages was poor even among subjects who fixated them, but, as in the studies by Fischer et al. (1989) and Krugman et al. (1994), there was a positive correlation between fixation time and masked recall performance.

Finally, Peterson et al. (2010) found that American adolescents viewed Canadian-style cigarette warnings, containing graphic images (e.g., of diseased tissue) and novel text warnings, for about 2.5 times as long as traditional, American warnings (including only text delivering the Surgeon General’s warning). Subjects also recalled the graphic messages more accurately in a subsequent memory test. Strasser et al. (2012) observed similar responses to graphic warnings on tobacco products among adult, American smokers.

Overall, then, the data seem quite clear that small, text-based warnings on advertisements receive little visual attention and are poorly recalled. However, by manipulating the salience (and the novelty) of such ads by, e.g., adding graphic images, attention and memory may be improved^8^.

### DYNAMIC MEDIA

Recent research has expanded beyond the realm of print advertising to examine eye movements when viewing ads presented via dynamic media, including websites and TV. While print advertisements can only use static cues, websites and TV also afford advertisers the opportunity to use sound and motion to guide viewers’ attention. Research that specifically examines viewers’ responses to dynamic media is essential for developing a complete understanding of the effects of sound and motion on attentional capture, memory, and preference. Several important findings regarding eye movements when viewing dynamic media are reviewed below.

#### Television advertisements

While research using eye tracking to examine the effectiveness of TV ads in capturing visual attention and affecting recall is relatively limited at this time, several interesting and potentially useful findings have nonetheless emerged from this literature (see also Wedel and Pieters, 2008a for a review).

First, in one early line of research, d’Ydewalle and colleagues (d’Ydewalle et al., 1988; d’Ydewalle and Tamsin, 1993) measured attention to and subsequent memory for advertisements appearing on billboards at a soccer field during a televised game. In both studies, subjects viewing the game on video spent less than 4% of the total time fixating the billboards. Perhaps unsurprisingly, given how little time was spent inspecting the ads, d’Ydewalle and Tamsin (1993) found that subjects recalled on average only 1.2 brands out of the 42 that were presented and were at chance for brand recognition. Thus, TV ads that are embedded within the primary content of a sporting event may not attract substantial visual attention or lead to strong memory representations of the advertised brand.

Other research has analyzed visual attention to more standard TV ads, typically presented during commercial breaks and interspersed with the primary content. Brasel and Gips (2008b) compared viewing behavior for TV shows and commercials. They found, first, that viewers exhibited a strong tendency to fixate near the center of the screen when viewing both kinds of content. They also conducted a frame-by-frame analysis of variability in fixation locations across subjects and found that variability was higher when viewing commercials than when viewing the primary program. Furthermore, variability of fixation locations was particularly high when the commercials contained brand elements. Finally, familiarity with a given commercial (manipulated by presenting it several times over the course of an experimental session) was also linked with increased variability of fixation locations. Brasel and Gips speculated that lack of engagement with the ad, driven by repeated presentations, could, perhaps explain the tendency for subjects’ eyes to wander more widely in later exposures to the ad.

Two studies by Teixeira and colleagues also examined variability in fixation locations across subjects, this time in connection with ad avoidance. Critically, if viewers do not wish to view TV ads (and video-based ads more broadly), they are often able to avoid them entirely, by muting them, temporarily turning off the device, or even blocking or skipping the commercials. The topic of ad avoidance is, consequently, an important one in the domain of TV advertising. Teixeira et al. (2010) found that higher variability in fixation locations across subjects predicted greater ad skipping. They suggested that high variability may indicate a failure, on the part of the advertiser, to sufficiently shape viewers’ engagement with the advertisement and guide attention to key aspects of the scene from one moment to the next. In addition, they found that the sustained presence of a central brand element on the screen predicted ad skipping^9^. However, brand “pulsing,” a strategy wherein the brand is shown for the same duration overall, but for shorter intervals each time, was found to ameliorate this effect. To explain this finding, Teixeira et al. speculated that pulsing, unlike the sustained, central presence of the brand, may leave the narrative of the commercial relatively intact, thus supporting effective guidance of viewers’ visual attention and preventing ad skipping.

Building up on these findings, Teixeira et al. (2012) examined the relationships among emotion, as measured by viewers’ facial expressions, variability in fixation locations, and commercial skipping^10^. They found that measures of apparent joy and surprise were linked with reduced variability in fixation locations across subjects. These emotions, in addition, were found to reduce ad skipping, both via a direct route (when controlling for fixation concentration effects) and via an indirect route, by concentrating fixation locations across viewers.

Quite recently, Brasel and Gips (2013) investigated the effect of subtitles on visual attention to and memory for ads. They found that same-language subtitles attracted visual attention, as subjects spent a greater percentage of frames looking at the subtitle region when subtitles were present than when they were absent. In addition, same-language subtitles also improved recall for the brand and for verbal information that was presented redundantly (i.e., both vocally and within the subtitles). Subtitles did not improve all aspects of memory, however: indeed, they decreased recall of information presented only visually, leading to reduced memory for brands that were not verbally named (and were therefore not included in the subtitles). The eye-tracking data and the memory data were collected from different subject groups, however, so it is not possible to correlate a given subject’s fixations on subtitles with subsequent recall performance.

Finally, Janiszewski and Warlop (1993) found evidence that attention to ads may be improved via a conditioning procedure. In the study, TV commercials were always presented in a specific order such that a conditioned stimulus (clip of the soda being advertised) always preceded an unconditioned stimulus (a clip of an enjoyable activity). This conditioning procedure led to increased (and more rapid) attention to the conditioned brand during subsequent exposure, suggesting that associative learning about a given brand can enhance attention to that brand.

In summary, research on TV ad viewing suggests, first, that embedded advertisements, in the form of billboards appearing during sporting events, may not be effective in capturing visual attention or influencing subsequent memory (d’Ydewalle et al., 1988; d’Ydewalle and Tamsin, 1993). When considering more traditional TV commercials, in which ads are interleaved with the primary content during commercial breaks, ad skipping is a central concern. Interestingly, when fixation locations are quite variable across subjects, more frequent ad skipping occurs (Teixeira et al., 2010), perhaps suggesting a lack of engagement with the narrative of the ad. Measures of joy and surprise are linked with more homogeneous viewing behavior across subjects and reduced brand skipping (Teixeira et al., 2012). In contrast, repeated exposures to an ad lead to increased variability in fixation locations across subjects (Brasel and Gips, 2008b). Including subtitles with TV ads is also associated with improved memory for certain kinds of information presented in the ads (Brasel and Gips, 2013). Finally, conditioning procedures can increase attention to brand elements in TV commercials (Janiszewski and Warlop, 1993).

#### Internet advertisements

As in TV advertising, ad avoidance is a topic of considerable interest in the domain of Internet advertising. Unlike most TV ads, banner and “skyscraper” ads (i.e., vertical banners) that appear on websites must often compete directly with surrounding editorial content for visual attention (see Drèze and Zufryden, 2000). As will be discussed below, viewers are thought to routinely avoid such ads when viewing websites, a phenomenon known as “banner blindness” (Benway, 1998, 1999; see also Owens et al., 2011 for similar findings regarding text ads). Several lines of research have manipulated the location, animation, onset, and relevance of Internet ads, simultaneously recording viewers’ eye movements to determine when the ads capture visual attention and when “banner blindness” takes place.

In one early study of eye movements during Internet search, Drèze and Hussherr (2003) found that subjects searching web sites fixated just under half of the banner ads presented. Since the probability of fixation was less than one would predict on the basis of ad size and location alone, Drèze and Hussherr concluded that viewers were able to identify banner ads in the visual periphery and, subsequently, intentionally avoid fixating them. Additionally, only 46.9% of subjects remembered seeing any banner ads during the experiment, and a recognition memory test revealed that subjects could not accurately discriminate ads that had been present on the website from foils that had never appeared.

Since certain Internet ad locations are consistent and thus predictable, however, users may not need to identify ads in the periphery in order to avoid them, but rather may be able to learn where they tend to appear and simply avoid fixating those locations. Lapa (2007) provided evidence that viewers do, in fact, learn the locations of banner ads over time and sometimes use this information to avoid fixating them. However, Burke et al. (2005) found that even when ad locations were not predictable, subjects only fixated the banners in 11.7% trials^11^. This suggests, as Drèze and Hussherr (2003) proposed, that subjects are, indeed, also able to recognize banner ads in peripheral vision and avoid fixating them.

While it appears that Internet ads may receive little attention in general, certain factors may be manipulated with the aim of attracting or holding viewers’ attention: these include location, animation, onset, and relevance. Kuisma et al. (2010) manipulated both ad location (horizontal, banner ads on the top of the display vs. vertical, “skyscraper” ads on the right side of the display) and animation (both static, both animated, or one of each). There was a main effect of ad location, such that more fixations landed on the skyscraper ad on the right side of the display than on the banner ads along the top. Animation was also found to increase fixations on skyscraper ads and decrease fixations on banner ads. Furthermore, including multiple animated advertisements resulted in fewer fixations on the ads than including only a single animated ad. Somewhat surprisingly, recognition memory results did not mirror the eye movement data. Rather, animation increased recognition memory for banner ads, but had no effect on the recognition memory for skyscraper ads. Findings on the relationships among memory, animation, and visual attention to Internet ads become even less clear when we consider the results of Burke et al. (2005), who found that memory (though very poor overall) was better for static banner ads than animated ones.

In a study similar to that of Kuisma et al. (2010), Simola et al. (2011) also manipulated both location (banner, skyscraper) and animation (both static, both animated, one of each), but additionally included different ad onset delays from 0 to 12 s. Consistent with the findings of Kuisma et al. (2010), they reported that animation increased attention to the skyscraper ads to the right of the text (especially when one ad was animated and the other remained static), and that the skyscraper ad was fixated more often and for longer than was the banner ad above the text. They also found that abrupt onset captured attention, as ads that appeared abruptly were fixated more often, though this effect was modulated by ad location, with skyscraper ads in close proximity to the text capturing attention more immediately, and banners located in the periphery capturing attention less quickly (see also Day et al., 2006 for evidence that even without capturing overt attention, ads flashing in the periphery can increase arousal and result in more efficient primary task performance).

Extending these findings, Simola et al. (2011) varied the task (reading for comprehension vs. browsing according to subjects’ own interests) and found that subjects were more likely to view the ads and looked at them for longer during browsing than during reading for comprehension, thus providing evidence that a user’s goals can exert “a strong top-down influence on attentional allocation” (p. 189) during online processing of information and ads. Additionally, during browsing, they found a correlation between ad onset and first fixation time for ads at both locations. However, in the reading task, there was only a correlation for the ad to the right of the text (which was in close proximity to the ends of the lines of text) and not to the peripheral banner ad, suggesting that users can selectively allocate attention to the task-relevant portions of the screen. Critically, in both tasks, self-reports of attention were correlated with actual eye movement data, such that participants who reported attention to ads also looked at the ads more often and for longer periods of time. This led Simola et al. to suggest that attentional capture by ads is related to overt rather than covert attention, a conclusion that seemingly runs counter to the studies suggesting that ads are recognized peripherally via covert attention (e.g., Drèze and Hussherr, 2003; Burke et al., 2005; Day et al., 2006).

Hamborg et al. (2012) examined the time course of attention to banner ads when subjects were given a primary task requiring that they extract information from an accompanying article. Significantly more subjects looked at a continuously animated than a static banner ad, in seeming contrast to some of the findings described above. Interestingly, these banner ads also attracted most fixations near the beginning or end of the primary task, suggesting that bottom-up salience may be more likely to interfere with top-down processing during these early and late periods of information search (see also Wang and Day, 2007). More details about the animated ads than the static ads were also recalled in a subsequent memory test.

Finally, some research has manipulated relevance of the ad to the subject’s task as well as the relationship between the ad and the editorial content. Lapa (2007) manipulated ad relevance by including ads that were either related or unrelated to the subject’s search task. He found that relevance did not influence ad viewing time, suggesting that users may assume banner ads to be irrelevant to their goals and the primary content. Relatedly, Hervet et al. (2011) found that congruency between text ads and surrounding web page content did not influence fixation probability or total viewing time on the ads, though congruent ads were remembered better than incongruent ones^12^.

In summary, viewers may tend to avoid fixating advertisements on websites, both by identifying them peripherally (Drèze and Hussherr, 2003; Burke et al., 2005) and by learning the locations in which they are likely to appear (Lapa, 2007). Some evidence also suggests that skyscraper ads, presented to the right of the primary content, are more likely to be fixated across a variety of tasks than are banner ads, presented on top of the primary text (Kuisma et al., 2010; Simola et al., 2011). Furthermore, the likelihood of fixating such skyscraper ads may be increased if they are animated (Kuisma et al., 2010; Simola et al., 2011) or appear suddenly (Simola et al., 2011). Effects of animating banner ads, however, are somewhat less clear (compare Hamborg et al., 2012 with Simola et al., 2011 and Kuisma et al., 2010). A mixed pattern of findings has also been reported concerning the relations among memory, animation, and eye movements when viewing Internet ads. In general, however, the data indicate that memory for Internet ads is rather poor (Drèze and Hussherr, 2003; Burke et al., 2005). The relevance of Internet ads (Lapa, 2007) or their relationship with surrounding content (Hervet et al., 2011) do not appear to affect ad viewing, suggesting that users may assume that such ads will be irrelevant to their primary goals. Finally, some evidence suggests that when viewers are engaged in a primary task, they are more likely to view banner ads near the beginning or the end of this task, when they may be more susceptible to bottom-up influences on oculomotor behavior (Wang and Day, 2007; Hamborg et al., 2012).

## CONCLUSION

In this article, we reviewed critical findings on eye movements when viewing advertisements, including in print, on TV, and on websites. A number of factors were found to guide eye movements when viewing prints ads, ranging from basic visual properties of advertisements (e.g., size and color), to social cues (e.g., the direction of a model’s gaze), to the goals of the viewer. The literature regarding warning labels on tobacco and alcohol ads revealed that the plain, black-and-white text warnings currently used in the United States draw little visual attention and are often forgotten. However, manipulating the visual salience (and novelty) of these warnings – by, for example, including graphic images – improved both visual attention to and memory for such warnings. Research on ads in dynamic media has also produced several noteworthy findings, revealing, for example, that subjects appear to avoid viewing banner ads in some cases, using both peripheral processing and canonical ad locations as cues. Across multiple domains, eye movement measures were often (though not always) found to predict subsequent memory for the advertised product, warning, or brand.

Although a substantial body of research has now been produced on eye movements while viewing advertisements, several avenues remain largely unexplored. First, relatively little is known about the guidance of eye movements when viewing dynamic, video-based ads (but see Itti, 2005 for a model of bottom-up effects on dynamic scene viewing). We expect that this will be an important area for future research to examine in greater depth. The relationships among eye movements, memory, and preference are also ripe for further investigation. The potentially complex causal relationships among these variables are of considerable theoretical interest^13^. Such research could also be quite useful from an applied perspective. For example, it would be helpful to determine how or whether specific eye movement measures might predict memory for a brand or product over an extended period of time (e.g., multiple days or weeks). As was noted above, tracking eye movements seems less likely to bias subsequent measures (such as product recall) than does soliciting verbal responses from subjects. Therefore, if eye movements are indeed a robust predictor of brand memory over some duration, this may be very helpful to applied researchers.

Several methodological approaches may also prove useful. First, though the point may seem rather a minor one, we strongly believe that settling on a common, codified set of terms to refer to the same, underlying eye movement measures (e.g., the total duration spent viewing a given element within a trial) will enable findings to be shared and compared much more efficiently across laboratories in the upcoming years. At present, the terminology used for such measures appears to be somewhat variable in the advertising literature. 

Second, the gaze-contingent display change paradigm (McConkie and Rayner, 1975; Rayner, 1975) may prove useful in future research. As was noted above, this approach, which consists in dynamically updating the display based on the eye movements of the viewer, has been quite useful in research on reading, visual decision-making, etc., allowing us to investigate topics such as parafoveal preview and the perceptual span in detail. The technique is useful because it affords experimenters precise control over subjects’ visual input, based on current eye position, while allowing subjects to inspect the scene freely. Gaze-contingent designs could, we believe, take on an important role in upcoming research on eye movements when viewing advertisements.

Third, and most broadly, further controlled, experimental designs could be used in future research to complement some existing correlational findings. A number of important studies in the field have used an approach that is at least partly correlational, presenting viewers with an assortment of real advertisements that vary naturally along dimensions of interest (e.g., the size of each ad element) and then measuring associated eye movement variables. This approach has advantages: notably, ecological validity is high. However, confounds are also a risk in such studies^14^. Therefore, it would be useful to determine if experimental studies, requiring systematic manipulation of independent variables of interest, will produce consistent results.

Finally, compared with the literature concerning eye movements in reading, scene perception, and visual search, it seems that research on eye movements while looking at advertisements is in its infancy. Consequently, a large number of interesting and useful avenues of research (of which only a few are mentioned above) remain available for future researchers to explore.

## Conflict of Interest Statement

The authors declare that the research was conducted in the absence of any commercial or financial relationships that could be construed as a potential conflict of interest.

## Footnotes

1 Suppose, for instance, that two draft versions of an ad were created and that one was consistently viewed for longer than the other. If the ads differed in several respects (pictorial, headline, etc.), an eye tracking experiment could efficiently reveal which element of the favored (or, at least, longer-viewed) ad was driving the effect. This information could then be used to inform the creation of new ads. As another example, suppose that behavioral experiments revealed that the inclusion of a particular new element in an ad – a line of text, for example, or a “packshot” showing the product – failed to increase memory and preference for the brand or product in question. Eye tracking could reveal whether the element was viewed (but, presumably, deemed unpersuasive) or simply never fixated. This, in turn, could provide useful clues about how the element should be revised, e.g., by changing its message or simply making it more visually salient (Lohse, 1997).

2 Please note that some important topics concerning eye movements and marketing lie beyond the purview of this article. For example, we do not cover point-of-purchase marketing here (e.g., consumer responses to supermarket shelf displays). However, this is an active area of research (see Wedel and Pieters, 2008a; Glaholt and Reingold, 2011; Orquin and Mueller Loose, 2013 for relevant reviews). The topic of roadside advertising and potential attendant distraction, while clearly a matter of great importance, is also beyond the scope of the present article.

3 Many complexities of the system are necessarily omitted from this short review. For example, the FEF also have direct projections to the premotor areas of the brainstem that are not relayed through the SC (Gaymard et al., 1998).

4 However, it should be noted that the particular nature of the memory test used here, in which subjects had to identify the advertised brand based on a pixilated version of the ad, seems likely to confer a relative advantage on the brand element when compared with other components. Note, for instance, that the body text was not easily resolvable from the pixilated version of the ad. Thus, further examinations should attempt replicate this result using different types of recall tests.

5 There is a typo in Table 6 of the chapter by Radach et al. (2003) suggesting that, in Experiment 2, the explicit ads were liked better and rated as more interesting. However, the main body of the text (with which the table conflicts) is correct in claiming that in both Experiment 1 and Experiment 2 the implicit ads were liked better and rated as more interesting (R. Radach, personal communication, October 17, 2013).

6 More specifically, the text was viewed longer and more often than the picture in this study for both critical and non-critical ads. For one of the ad types only (depicting cars), however, the text advantage was greater when those ads were critical than when they were not.

7 Reading time was calculated as the sum of all fixations with durations of 100 ms or more, not by a qualitative assessment of the eye movement patterns in relation to the text. Individual fixations shorter than 100 ms were counted in looking time, but not reading time. If a subject made no fixations over 100 ms in duration, they were deemed not to have read. A more detailed investigation of the eye movement data was not included.

8 For an additional example of research using eye tracking to examine the effectiveness of public health messages, see O’Malley et al. (2012), which concerns visual attention when viewing osteoporosis prevention ads.

9 See Brasel and Gips (2008a), however, for results suggesting that a central brand element may be beneficial for memory for brands viewed in fast-forwarded commercials.

10 The ads tested in this study were, in fact, Internet ads. However, they are included in this section because they represent video-based ads and are similar in form to television advertisements.

11 This 11.7% estimate is an upper bound since in 70% of these trials, the ad was fixated following the first eye movement and in 54% of this subset, the ad actually appeared in the location of the first fixation after the eyes had already moved.

12 As discussed above, however, Simola et al. (2013) found that newspaper ads that were semantically incongruent with primary content received more attention than those that were semantically congruent in second-pass viewing.

13 Note that related issues have been addressed in some detail in the visual decision-making literature (see, e.g., Glaholt and Reingold, 2011).

14 For example, suppose that brands that sold particularly intriguing products also tended, on average, to use large pictures in their advertisements. If we found longer gaze durations associated with larger pictures, then, it may be attributable to underlying characteristics of the product rather than the size of the picture.
